# The Subcortical Cocktail Problem; Mixed Signals from the Subthalamic Nucleus and Substantia Nigra

**DOI:** 10.1371/journal.pone.0120572

**Published:** 2015-03-20

**Authors:** Gilles de Hollander, Max C. Keuken, Birte U. Forstmann

**Affiliations:** 1 Amsterdam Brain & Cognition Center, University of Amsterdam, Amsterdam, Netherlands; 2 Max Planck Institute for Human Cognitive and Brain Sciences, Leipzig, Germany; Centre Hospitalier Universitaire Vaudois Lausanne - CHUV, UNIL, SWITZERLAND

## Abstract

The subthalamic nucleus and the directly adjacent substantia nigra are small and important structures in the basal ganglia. Functional magnetic resonance imaging studies have shown that the subthalamic nucleus and substantia nigra are selectively involved in response inhibition, conflict processing, and adjusting global and selective response thresholds. However, imaging these nuclei is complex, because they are in such close proximity, they can vary in location, and are very small relative to the resolution of most fMRI sequences. Here, we investigated the consistency in localization of these nuclei in BOLD fMRI studies, comparing reported coordinates with probabilistic atlas maps of young human participants derived from ultra-high resolution 7T MRI scanning. We show that the fMRI signal reported in previous studies is likely not unequivocally arising from the subthalamic nucleus but represents a mixture of subthalamic nucleus, substantia nigra, and surrounding tissue. Using a simulation study, we also tested to what extent spatial smoothing, often used in fMRI preprocessing pipelines, influences the mixture of BOLD signals. We propose concrete steps how to analyze fMRI BOLD data to allow inferences about the functional role of small subcortical nuclei like the subthalamic nucleus and substantia nigra.

## Introduction

The subthalamic nucleus (STN) and substantia nigra (SN) are small subcortical nuclei in the basal ganglia [1]. Many cognitive neuroscience studies using BOLD functional magnetic resonance imaging (fMRI) have shown that the STN and SN are involved in a range of tasks such as response inhibition [2–4], conflict processing [5], force production [6,7], working memory [8,9], and the adjustment of global and selective response thresholds [10] (Table 1). While several studies have shown the involvement of either the STN or the SN in tasks such as the stop-signal task or Simon task, several neurocomputational models make distinct functional predictions for these nuclei (e.g.[11,12]). For instance, Frank et al. [12,13] propose that the STN acts as a general brake, whereas the SN, through the release of dopamine, is activated by the correct response and inhibits the incorrect response. However, the small size and close proximity of the STN to the SN, as well as to the surrounding brain structures, frustrates precise localization and makes it challenging to attribute the BOLD signal to either STN or SN, respectively. A common procedure is to place a box of 10x10x10 mm on a center coordinate in the STN and extract the mean BOLD fMRI signal in this box, to estimate the signal change in the STN [2,14,15]. Note that the volume of such a box is about 7.5 times larger than the average STN volume reported in the literature (1000 mm^3^ as compared to a weighted average of 119.88 mm^3^ and a weighted median of 131.75 mm^3^ [16–29]. In combination with often-used smoothing procedures, the signals originating from the STN and SN will get mixed, making it difficult to unequivocally attribute signal to either structure [30,31].

**Table 1:** Literature overview of BOLD fMRI STN and SN studies.

Author	Task	Age	Tesla	fMRI resolution (mm)	voxel size (mm^3^)	FWHM (mm)	Structure	Definition of ROI	MNI peak coordinate		
									x	y	z
[53]	Feedback-driven classification-learning	20–33	3	3.125x3.125x6*	58.59	8	SN/VTA	Search space: 15mm3 sphere (0,-15,-9)	-6	-21	-9
[14]	Stop-signal paradigm	29.2 (4.5)	3	3.125x3.125x4	39.06	5	STN	ROI: 10mm3 box (10,-15,-5)	8	-20	-4
							STN		6	-18	-2
							STN		10	-14	-4
							STN		14	-18	-4
		23.8 (3.7)		1.56x1.56x3	7.30	2	STN	M.S. TSE sequence	N.S.	N.S.	N.S.
[54]	Stop-signal paradigm	28.1 (4.1)	3	3.125x3.125x4	39.06	5	STN	ROI: 10mm3 box (10,-15,-5)	6	-18	-4
							STN		8	-16	-6
							STN		14	-8	-4
							STN		10	-14	-4
[36]	Resting State	63.2 (8.7)	3	3x3x3*	27	5	STN	M.S. EPI sequence of 2 axial slices (z: −6 & −8)	-12	-14	-8
							STN		-14	-8	-6
[5]	Counting stroop	10.2 (0.8)	1,5	3.36x3.36x4	45.16	12	SN	Talairach atlas	-11,92	-19,31	-8,07
	Go/No-go		1,5	3.36x3.36x4	45.16	12	SN	Talairach atlas	-2,28	-12,25	-13,42
[55]	Automated four-digit finger sequence	25.8 (4.7)	3	3.6x3.6x3.6	46.66	8	SN	Talairach atlas	-2,32	-19,15	-18,34
							SN	Talairach atlas	1,15	-18,85	-15,57
[4]	Stop-signal paradigm	24.5 (n.s.)	3	3x3x3.3*	27	4	SN	Sig. voxels in the SN region	10	-22	-20
			3	3x3x3	27	8	SN	Sig. voxels in the SN region	12	-24	-14
							STN	Sig. voxels in the STN region	10	-16	-2
[56]	Visual discrimination	26 (n.s.)	3	2x2x3.6	14.4	6	SN	Visual inspection on mean PD sequence	8	-20	-14
							SN		-6	-18	-16
[57]	Resting State	29.9 (n.s.)	3	1.56x1.56x3	7.3	3	STN	Talairach atlas	N.S.	N.S.	N.S.
[58]	Checker board	18–35	1,5	N.S.xN.S.x3*	-	8	SN		-10	-16	-10
	Cognitive color-word stroop		1,5	N.S.xN.S.x3*	-		SN	ROI: 8mm sphere on peak voxel	-12	-2	-8
[59]	Visual oddball	23.9 (4.2)	3	3x3x3.3*	29.7	4	SN/VTA	M.S. MT sequence	8	-20	-18
							SN/VTA	M.S. MT sequence	12	-18	-20
[37]	Visual oddball	65.3 (6.3)	3	3x3x3.3*	29.7	4	SN/VTA	M.S. MT sequence	0	-14	-12
[60]	Slot machine	33.7(1.8)	3	3.1x3.1x3	28.83	10	SN/VTA	Coordinates of Duzel et al. 2008	-8	-20	-14
						4	SN/VTA		-8	-18	-18
							SN/VTA		12	-16	-12
							SN/VTA		-6	-18	-16
[61]	Three-stage retrospective revaluation	25 (5)	3	3.1x3.1x5*	48.05	8	SN	Pickatlas	14	-20	-5
							SN		-10	-18	-8
[15]	Motor task switching	25.2 (n.s.) / 67.9 (n.s.)	3	2.5x2.5x2.83*	17.69	10	STN	ROI: 10mm3 box (10,-15,-5)	10	-15	-5
[62]	AX-CPT	20–53	3	1.5x1.5x1.9	4.28	3	SN/VTA	Talairach atlas	-5,51	-11,1	-12,36
[63]	Sequential decision making	19–53	3	1.5x1.5x1.9	4.28	3	SN/VTA	M.S. PD sequence	-1,32	15,3	-17,28^±^
							SN/VTA	M.S. PD sequence	-3,5	17,32	-18,57^±^
							SN	M.S. PD sequence	13,75	22,59	-20,5^±^
[3]	Stop-signal paradigm	22–45	3	3.4x3.4x4	46.24	10	STN	AAL atlas	N.S.	N.S.	N.S.
[64]	Perceptual decision making	23.9 (n.s.)	3	3x3x3*	27	8	STN	N.S.	-15	-18	0
[65]	Perceptual decision making	25.3 (n.s.)	3	3x3x3*	27	8	STN	ROI: 10mm3 box (10,-15,-5)	10	-15	-5
[66]	Go/No-go	23 (1.72)	3	1.5x1.5x1.5	3.38	6	SN/VTA	M.S. MT sequence	8	-9	-10
							SN/VTA	M.S. MT sequence	-12	-19	-7
[67]	Go/No-go	23.3 (5)	3	1.5x1.5x1.5	3.38	6	SN/VTA	M.S. MT sequence	12	-18	-10
							SN/VTA	M.S. MT sequence	-7	-22	-14
							SN/VTA	M.S. MT sequence	7	-20	15^±^
[68]	Simon task	23 (3.9)	3	N.S.xN.S.x3.7*	-	8	SN/STN	ROI: 12 mm sphere (-10,-15,-5)	16	-8	-10
							STN		-14	-12	-6
[69]	Stop-signal paradigm	27.6 (5.5)	3	3.4x3.4x4	46.24	8	STN	Pickatlas	3	-25	-2
							STN		6	-13	-5
[70]	Simon task / Stop-signal paradigm	23.6 (n.s.)	3	2.3x2.3x3.3*	17.46	N.S.	STN	Anatomical ROI centered on 8,-9,-11	8	-9	-11
[71]	Nonaversive differential conditioning	23.3 (n.s.)	1,5	3x3x5	45	6	SN	Talairach atlas	-8,37	-15,78	-15,73
							SN	Talairach atlas	10,69	-24,87	-11,89
							SN	Talairach atlas	-8,34	-18,59	-9,99
							SN	Talairach atlas	10,67	-22,06	-17,63
[72]	Reward anticipation paradigm	25 (2.9)	3	3.5x3.5x3.5	42.88	6	SN/VTA	N.S.	9	-18	-18
							SN/VTA		9	-12	-18
							SN/VTA		15	-15	-9
[73]	Reward anticipation paradigm	24.7 (2.1)	3	1.5x1.5x2	4.5	3	SN	ROI: 2mm sphere on peak voxel	9	-19	-14
							SN		10	-19	-15
							SN		12	-17	-8
[74]	Spatial attention	21.7 (3.2)	3	3x3x3	27	6	STN	ROI: 2mm sphere on peak voxel	8	-16	-6
							SN/VTA		4	-14	-12
							SN/VTA		-2	-16	-14
							SN/VTA		2	-20	-16
							SN/VTA		-4	-14	-12
							SN/VTA		4	-12	-12
[75]	Complex motor sequence	22.9 (3.9)	3	1.5x1.5x2.5	5.63	10	STN	Atlas by Yelnik et al. 2003	-13,62	-16,2	-4,57
							STN		-11,53	-12,11	-7,08
							STN		11,71	-12,05	-9,44
							STN		-11,53	-12,11	-7,08
							STN		15,94	-11,88	-7,26
							STN		11,71	-12,05	-9,44
[76]	Counting stroop task	10.2 (1.3)	1,5	N.S.xN.S.x4	-	N.S.	SN	-	-11,92	-19,31	-8,07
[77]	Motor task switching	24.5 (n.s.) / 25.3 (n.s.)	3	2.5x2.5x3.08*	19.25	10	STN	ROI: 10mm3 box (10,-15,-5)	8	-10	-8
							STN		-5	-10	-8
[10]	Task switching	23.4 (4.8)	1,5	4x4x4	64	8	STN	ROI: Forstmann et al. 2010 masks	N.S.	N.S.	N.S.
[78]	Complex motor	27.7 (2.4)	3	3.4x3.4x3.3*	38.15	6	STN	Talairach atlas	N.S.	N.S.	N.S.
[79]	Probability discount	26.6 (4.2)	3	2x2x3*	12	8	SN/VTA	Coordinates of Schott et al. 2006	6	-20	-10
							SN/VTA		-8	-16	-12
							SN/VTA		-10	-16	-12
[80]	Montreal card-sorting	23.4 (n.s.)	1,5	4.7x4.7x4.7	103.82	6	STN	Talairach atlas	-11,86	-24,32	-4,22
							STN		-9,81	-23,02	-13,35
							STN		14,03	-20,18	-7,31
[81]	Reward learning	26 (3)	3	3.1x3.1x5*	48.05	6	SN/VTA	N.S.	10	-8	-6
							SN/VTA		-4	-16	-6
							SN/VTA		-8	-20	-6
							SN/VTA		12	-22	-4
							SN/VTA		-8	-20	-8
							SN/VTA		14	-16	-6
							SN/VTA		8	-22	-8
[82]	Gambling	21.4 (n.s.)	3	3.28x3.28x3	32.28	8	STN	Talairach atlas	15,97	-18,05	-4,62
							SN		2,21	-18,78	-14,47
[83]	Force production	20–37	3	3.125x3.125x3	29.30	N.S.	STN	BGHAT template	-10,46	-14,14	-5,84
[84]	Stop-signal paradigm	22–45	3	3.4x3.4x4	46.24	6	STN	ROI: 10mm3 box (10,-15,-5)	-6	-21	-3
							STN		-6	-21	-3
							STN		9	-21	-6
							STN		-12	-12	-6
							STN		12	-12	-3
							STN		-12	-12	-3
							STN		12	-12	-3
							STN		-12	-15	-3
							STN		12	-15	-9
[85]	Resting state	26 (5)	3	3.3x3.9x4*	51.48	0	STN	N.S.	N.S.	N.S.	N.S.
							SN	N.S.	N.S.	N.S.	N.S.
[86]	Associative memory	n.s. / 18–31	1,5	3.13x3.13x6*	58.78	8	SN	M.S. MT sequence	N.S.	N.S.	N.S.
[87]	Delayed monetary incentive task	22.8 (1.5)	3	3.5x3.5x2	24.50	6	SN/VTA	M.S. PD sequence	-7	-23	-18
[88]	Face scene association learning	18–24	3	3.125x3.125xN.S.	-	8	SN/VTA	ROI: 10mm3 sphere on peak voxel Adock et al. 2006	3	-18	-12
[7]	Force production	20–35	3	3.125x3.125x3	29.30	5	STN	Talairach atlas	-10,46	-14,14	-5,84
[6]	Force production	21–35	3	3.125x3.125x5	48.83	N.S.	STN	Talairach atlas	N.S.	N.S.	N.S.
[89]	Force production	21–35	3	3.125x3.125x3	29.30	0	STN	Talairach atlas	-10,46	-14,14	-5,84
[38]	Resting state	55.3 (n.s.)	3	4x4x5	80	8	STN	ICA	9	-11	-3
							STN	Talairach atlas	-9	-12	-3
							SN	ICA	-9	-18	-12
[90]	Reward anticipation	22.9 (3)	1,5	3.13x3.13x6*	58.78	8	SN	Talairach atlas	7,44	-22.1	-15.97
							SN		11,04	-19,39	-12,83
[91]	Novelty	24.5 (4)	3	3x3x3	27	4	SN/VTA	Talairach atlas	5,28	-23,17	-15,83
							SN		14	-24,75	-10,22
							STN		-7,6	-11,65	-6,67
[8]	Working memory paradigm	33.1 (10.7) / 28.8 (7.3)	1,5	3.4x3.4x4	46.24	2	SN	Pickatlas	-8	-16	-12
							SN		8	-16	-14
[9]	Working memory updating	28 (4.4)	3	3x3x3	27	4	SN/VTA	M.S. MT sequence	10	-12	-12

**Fig 1:**
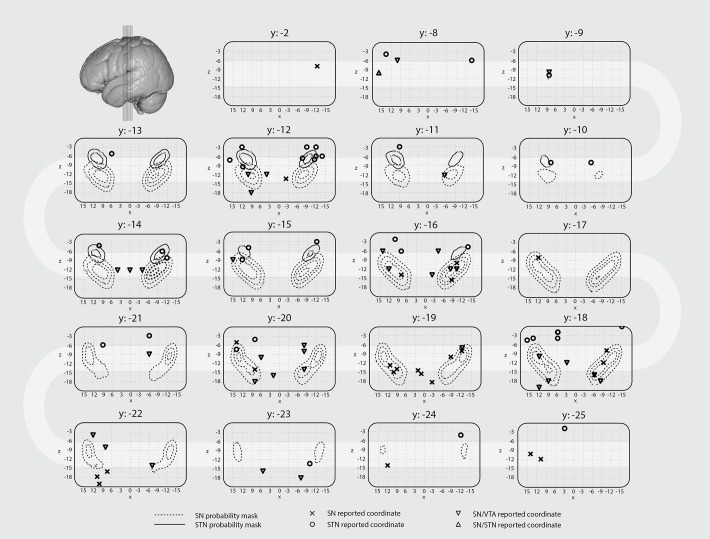
Location of individual peak coordinates of the STN and SN. Coronal slices in anterior to posterior direction are displayed together with functional coordinates of the STN, SN, SN/VTA, and SN/STN as reported in Table 1. Overlaid onto these coordinates is the probabilistic atlas of the STN and SN. The isolines reflect the percentage overlap across the 30 young subjects taken from Keuken et al. [28]. The outermost isolines reflects a 10% probability of containing the SN at the population level, the more inner lines represent 30%, 50%, and 70% probability of containing the SN. The outermost isolines for the STN reflects a probability of 20% containing the STN, the inner line represent 40% probability of containing the STN. The grid size corresponds to a voxel size of 3x3 mm. All coordinates are in MNI standard space.

M.S. Manual segmentation, TSE: Turbo spin echo, PD proton-density weighted, MT: Magnetization transfer,

* a slice gap was used,

±: coordinates not displayed in Fig. 1. The age is given in the mean years if provided, otherwise the range is given. N.S. not specified.

The goal of this study was to investigate the consistency of the coordinates found in fMRI studies on the STN and SN, summarize the methods employed in these studies, and assess the severity of the problems with localization and mixture of signals. In a first step, we conducted a comprehensive literature search to characterize the methods resulting in significant functional activation in the STN and SN. In a second step, the peak coordinates of the STN and SN derived from these studies were compared to the location of recently published probability STN and SN ultra-high resolution 7T MRI atlas [28]. Thirdly, using ultra-high resolution individual anatomical MRI masks, a simulation study was performed to test the influence of different smoothing kernels on the mixture of BOLD fMRI signals from both the STN and SN.

## Materials and Methods

### Selection of STN and SN BOLD fMRI studies

A comprehensive search for relevant neuroimaging studies in the field of BOLD fMRI studies including the STN and SN was carried out using Google scholar (http://scholar.google.com/). The main keywords utilized were ‘fMRI + substantia nigra’, ‘fMRI + SN’, ‘fMRI + subthalamic nucleus’, ‘fMRI + STN’, as well as all combinations of the aforementioned terms.

Based on the information contained in the abstracts of all the papers returned, empirical studies were selected to meet the following inclusion criteria: (1) Studies were published in peer-review English language journals between January 2000 and March 2014; (2) the studies used BOLD fMRI; (3) the studies reported a functional coordinate that could be attributed to either the SN or STN; and (4) the studies reported the location of activation as 3D coordinates in stereotactic space of Talairach or the Montreal Neurological Institute (MNI).

All empirical studies included were cross-referenced and all papers citing these empirical studies were searched, using the Google scholar citation index tool. The whole selection process was repeated for the newly obtained empirical papers until no new studies were found. This resulted in the inclusion of 52 papers (see Table 1).

All activation foci of the included studies that were originally reported in Talairach space were converted to the MNI stereotactic space using the Lancaster et al. transformation algorithm, which has been validated and shown to substantially reduce any bias between the two references spaces [32].

### Probabilistic ultra-high resolution 7T MRI atlas maps

For analysis of the comparison between STN and SN coordinates reported in the literature (see Table 1), we used previously reported ultra-high resolution 7T MRI probability maps [28]. The probability maps are based on 30 participants (14 females) with a mean age of 24.2 year (SD 2.4). The STN and SN masks were manually segmented by two raters for each individual on 7T zoomed multi-echo 3D FLASH MRI data with an isotropic voxel size of 0.5 mm [33]. Only voxels rated by both raters as belonging to the STN or SN were included in further analyses. Note that no differentiation between the SN pars compacta and the pars reticulate were made because the voxel resolution and used scan sequence did not allow for identification of the two subparts. The individual masks were then linearly registered to MNI standard space and combined to create a probabilistic atlas. For more information regarding the segmentation, MRI scanning sequence, and registration procedure see [28,34]. The structural data can be found on http://www.nitrc.org/projects/atag_mri_scans/ and on http://dx.doi.org/10.5061/dryad.fb41s. The probabilistic masks can be found on http://www.nitrc.org/projects/atag.

### Simulation study

A simulation study was performed to assess the amount of signal that originates from neighboring nuclei that can be introduced into a region of interest (ROI) by smoothing. Sixty STN and sixty SN masks (thirty masks in both hemispheres) from the ATAG (Atlas of The bAsal Ganglia) dataset [28] were used in a total of 60 simulations, all using one STN and one SN mask at a voxel resolution of 0.5 mm isotropic. It was assumed that every voxel in each mask contained a signal of unit strength. Then, smoothing kernels of different sizes were applied, and for every voxel and for every nucleus, the amount of signal in that voxel originating from that nucleus was determined. If the entire signal came from the same nucleus, the value was 1. If no signal from that nucleus reached that voxel, the value was 0. The sum of the signal strengths of the two nuclei in a voxel could never surpass 1.

We focused on the mixture of signal in the center voxel of both masks, to emulate a ROI study where the ROI would be placed in the best possible voxel according to the ground truth. This is a very optimistic scenario considering the difficulty of STN/SN localization as discussed earlier. For every center voxel, two quantities were computed:

$$mass_{SN}=\sum_{x}\sum_{y}\sum_{z}exp(-(\frac{\left(x-x_{com}\right)}{2\sigma^{2}}+\frac{\left(y-y_{com}\right)}{2\sigma^{2}}+\frac{\left(z-z_{com}\right)}{2\sigma^{2}})SN\_mask[x,y,z]$$

and

$$mass_{STN}=\sum_{x}\sum_{y}\sum_{z}exp(-(\frac{\left(x-x_{com}\right)}{2\sigma^{2}}+\frac{\left(y-y_{com}\right)}{2\sigma^{2}}+\frac{\left(z-z_{com}\right)}{2\sigma^{2}})STN\_mask[x,y,z]$$

corresponding to the amount of signal originating from the SN and the amount of signal originating from the STN.

[x_com_,y_com_,z_com_] is the coordinate of the center-of-mass of the mask-of-interest in millimeters.STN_mask[x,y,z] was either 1 or 0, corresponding to the coordinate [x,y,z] being in the STN or not, SN_mask[x,y,z] analogously for the SN. σ is the standard deviation of the Gaussian kernel, which can be calculated for a given FWHM (full width at half maximum) by using the following formula:

$$\sigma =\frac{FWHM}{2\sqrt{2\ln2}}$$

## Results

### Overview of functional MRI STN and SN studies

52 functional MRI studies were included in the present study (Table 1), published between 2003 and 2014. These studies employed for instance resting state, the stop-signal task, decision-making tasks including reward and outcome manipulations, and threshold adjustments in cognitive control tasks. Smoothing kernels ranged between 0 mm – 12 mm and data was collected on either 1.5 or 3T scanners. Note that not all studies report extensive methodological or procedural information, which limits the assessment of their anatomical specificity [35].

### STN and SN coordinates in MNI space compared to ultra-high resolution 7T atlas maps

The reported STN and SN-coordinates were compared to the center-of-mass coordinates of the previously published 7T MRI probabilistic masks [28]. Results are summarized in Table 2.

**Table 2:** Average deviation of reported coordinates from center of mass ATAG masks.

	N of reported coordinates (n of studies)	Distance in x	Distance in y	Distance in z	Total distance
**Left hemisphere**					
SN	12 (9)	1.0 (3.4)	0.5 (4.9)	0.6 (3.6)	5.6 (4.0)
SN/VTA	17 (11)	2.9 (2.8)	2.6 (11.8)	−0.3 (4.2)	9.1 (9.6)
STN	20 (12)	−0.9 (2.8)	−1.2 (4.4)	1.3 (2.8)	5.2 (3.3)
**Right hemisphere**					
SN	18 (10)	−0.9 (3.7)	−1.6 (11.1)	−1.8 (4.0)	8.9 (8.6)
SN/STN	1 (1)	5.5 (-)	8.2 (-)	2.2 (-)	10.1 (-)
SN/VTA	17 (11)	−2.6 (4.1)	−1.3 (4.5)	0.2 (3.8)	7.1 (2.6)
STN	17 (14)	−1.6 (2.9)	−3.7 (4.2)	3.0 (5.1)	7.3 (4.7)

Distance of reported MNI coordinates from the center of mass of the corresponding ATAG STN probabilistic mask (for STN coordinates) or ATAG SN probabilistic mask (for all other coordinates, “SN”, “SN/STN” and “SN/VTA”) in millimeters (standard deviation). A coordinate with a higher X-value lies more to the right. A coordinate with a higher Y-value lies more anterior. A coordinate with a higher Z-value lies more superior.

On average, reported STN activity coordinates lay 5.2 mm (left hemisphere, std. = 3.3) and 5.7 mm (right hemisphere, std. = 2.8) from the center-of-mass of the 7T MRI probabilistic mask. Several studies include older participants [15,36–38]). This might result in a mismatch between the reported coordinates and the probabilistic atlas because it is known that the STN shifts in lateral direction with age [29,39,40]. Note, however, that this lateral shift is smaller (on average 1.6 mm more lateral for elderly than for young participants [29]) compared to the standard deviation reported in the present study. The mismatch in location is predominantly observed in the dorsal-ventral and anterior-posterior direction such that the reported coordinates were on average 1.3 / 1.5 (left/right, std. = 2.8/2.5) mm more dorsal and 1.2 / 2.5 (left/right std. = 4.4/4.0) mm more anterior than the center-of-mass of the 7T probabilistic STN masks.

The left STN coordinates were on average 0.9 mm (std. = 2.8) more medial than the center-of-mask of the 7T MRI probabilistic mask, and the right STN coordinates lay on average 0.4 mm (std. = 3.2) more lateral. The average distance between the centers-of-mass of the SN and STN of the probabilistic masks is in the same order of magnitude as the distance between the average reported STN fMRI location and the actual STN center-of-mass (6.4 mm left, 6.7 mm right; std. = 0.7 / 0.7).

Reported SN activity coordinates were on average 5.6 (left hemisphere, std. = 4.0) and 8.9 (right hemisphere, std. = 8.6) mm away from the center of mass of the 7T MRI probabilistic mask. Shifts occurred in all three directions.

Fig. 1 shows coronal plots of the probabilistic maps with the reported coordinates rendered onto them. Only coordinates that lay within the MNI coordinate range of x: 18,-18 / z: 0,-21 / y: −2,-25 were plotted. The coordinates that fell outside of this range are marked in Table 1.

**Fig 2:**
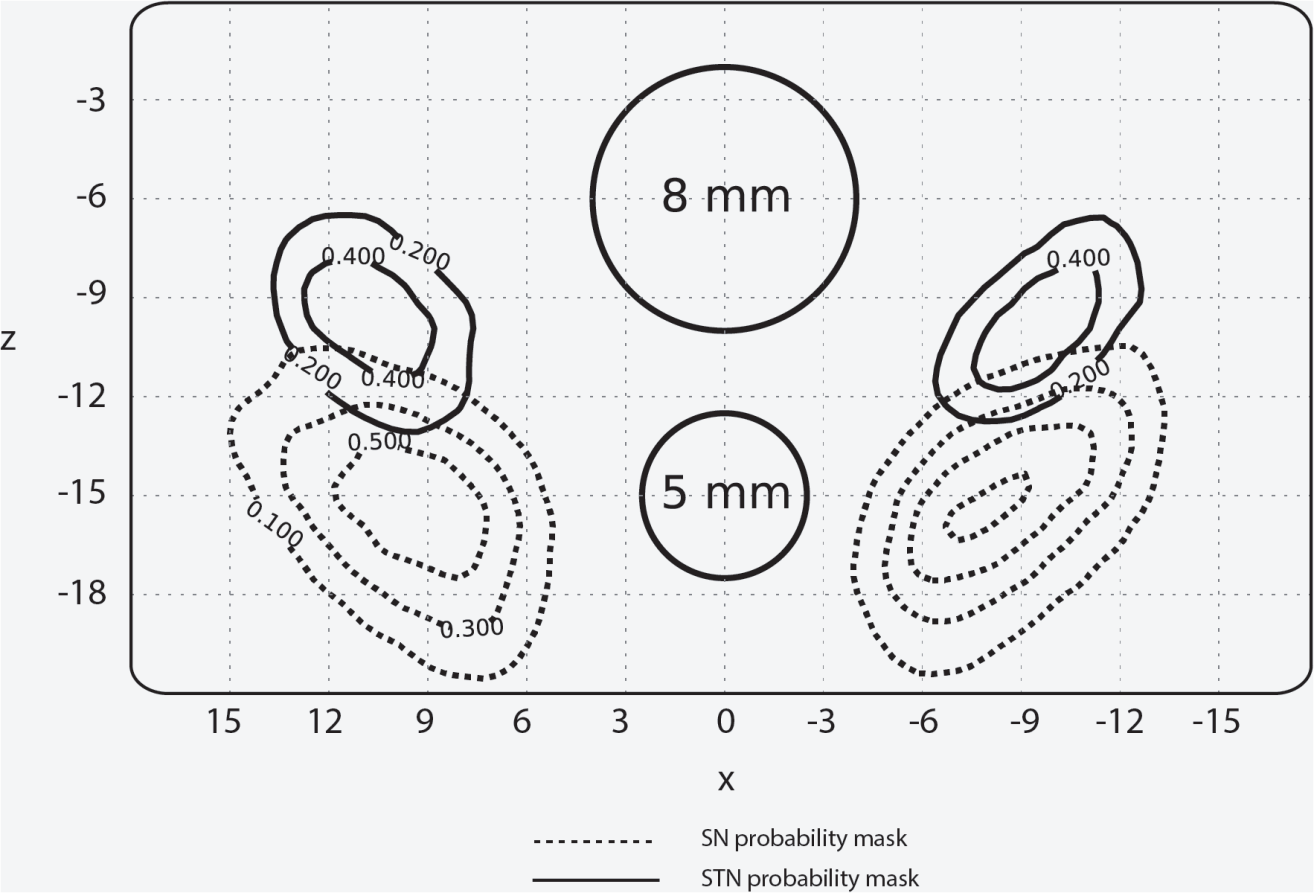
Relative size of a standard FWHM diameter compared to the STN and SN. A zoomed-in coronal slice (MNI y-coordinate: −14) showing the STN and SN. Two circles are shown in the middle to indicate the diameter of 2 frequently used FWHM smoothing kernels. The isolines reflect the percentage overlap across the 30 young subjects taken from Keuken et al.[28].

The average FWHM size of the smoothing kernel was 6.3 mm (median 6 mm). The 8 mm FWHM smoothing kernel was used most frequently (16 out of 52 studies: see Fig. 2 for the relative size of the kernels used compared to the STN and SN). There was no relationship between the nucleus of interest and the smoothing kernel used.

To test for any spatial biases introduced by smoothing, as reported by e.g. Sacchet et al., [41] for the nucleus accumbens, MNI coordinates were correlated with the size of the smoothing kernel employed. No correlations were found except for the SN (r(45) = .83, p <0.05, uncorrected), which lay more superior as a function of a larger smoothing kernel. The majority of studies (47 out of the 52) reported the voxel resolution. The voxel resolution was on average 34 mm^3^ (median 32 mm^3^, std. = 21 mm, range 3.4–103.8 mm).

### Simulation of effects of smoothing on subcortical fMRI activations

**Fig 3:**
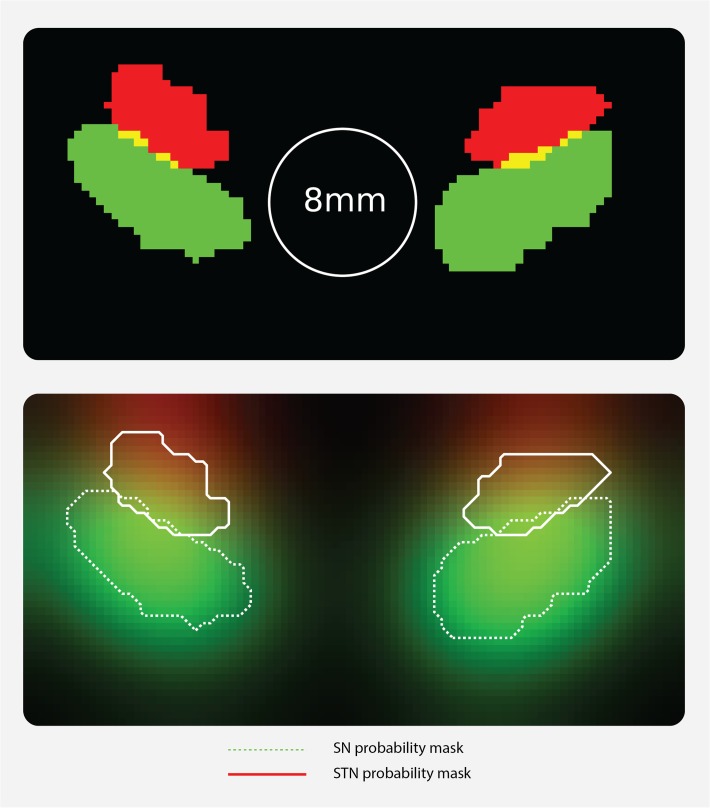
Illustration of effect of smoothing on mixture of BOLD signals between SN and STN. Four binary, individual masks are displayed of one representative participant smoothed with an 8 mm FWHM smoothing kernel.

**Fig 4:**
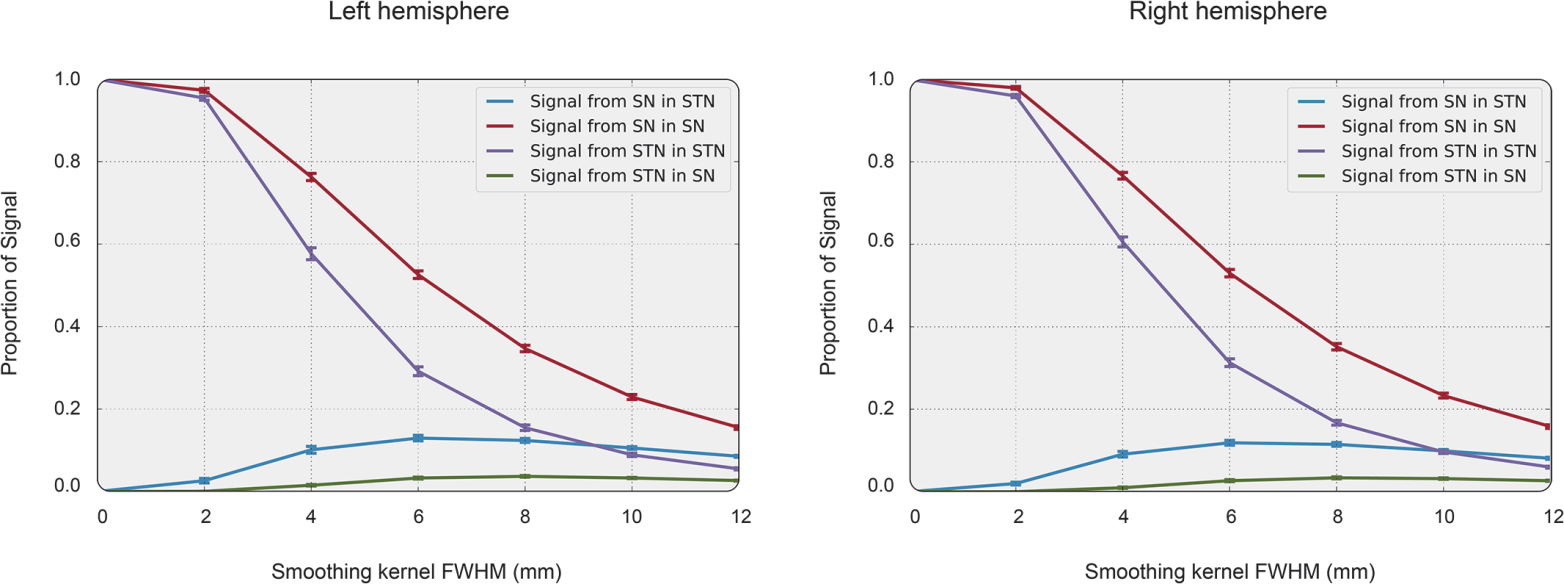
Simulation results: Effect of smoothing on mixture of BOLD signals between SN and STN. Summary of the smoothing simulation study. For both hemispheres, in 30 subjects taken from Keuken et al. [28], the effect of smoothing on the mixing of signals in STN and SN in their respective center voxels was estimated. The lines show the amount of signal for different source-destination pairs of STN and SN as a function of smoothing kernel size. When no smoothing is applied, all signal in the SN originates from SN and all signal in STN originates from the STN. When more smoothing is applied, the amount of signal originating from the nucleus that is measured sharply decreases, and within the STN the amount of signal from the SN becomes equal in size to the signal originating from the STN itself.

Fig. 3 qualitatively illustrates the effect of an 8 mm FWHM smoothing kernel on individual masks of the left and right STN and SN. The result shows that signal originating from each nucleus spreads widely, also across its neighbor’s boundaries (Fig. 4).

When no smoothing was applied, the signal in the center voxel of both SN and STN originated completely from the nucleus of its location. When a 4 mm FWHM smoothing kernel was used, 30% of the signal in the center voxel of the STN was found to originate from outside the STN and SN, while ten percent originated from the SN and 60% from the STN itself. With an often-used 8 mm FWHM smoothing kernel, 75% of the signal in the center voxel of the STN mask originated from outside the STN and SN. Ten percent originated from the SN and only 15% from the STN itself. For this simulation, the strength of the STN signal in the center voxel was taken to be similar in size to the signal originating from the SN. Note that the results would vary if different signal strengths in both nuclei were assumed. However, the precise ratios have little effect on this finding. Importantly, this simulation shows that with large smoothing kernels it becomes impossible to disentangle the origin of the measured signal, even when focusing on the most central voxel. In empirical fMRI studies this is an unlikely scenario, because the voxel resolution is at an average of 34 mm3, instead of the 0.064 mm3 (0.4 mm isotropic) used here. In sum, the mixing of signals is likely to be substantially worse in empirical fMRI studies compared to this simulation study.

## Discussion

In the present study we show that there is large variability in previously reported fMRI coordinates attributed to the STN and SN. We also show a discrepancy between individual coordinates of empirical studies and probabilistic atlas maps derived from ultra-high resolution 7T MRI [28]. The resolution of the fMRI sequences used in the studies we included was usually low compared to the size of the nuclei of interest. The average voxel resolution was 34 mm^3^ (median 32 mm^3^; std 21 mm^3^). Over the past years, the voxel resolution has also increased on 3T scanners. For all studies published since 2010 on 3T, mean voxel size was 22 mm^3^ (median 23 mm^3^; std 18 mm^3^). There is a significant correlation between year-of-publication and voxel size (r(115) = −0.31, p<0.001). However, half of the studies published in 2013 and later (n = 9) still use a coarse voxel resolution; on average they used a resolution of 24 mm^3^ (median 27 mm^3^, std 20 mm^3^), which results in only approximately 5 voxels covering the STN.

The simulation results reveal that when smoothing kernels of commonly used sizes are applied, the amount of signal from neighboring nuclei that get smoothed into a region of interest is of similar size as the signal from the region itself. This is particularly important when analyzing data from a small nucleus such as the STN, which borders the larger SN.

These results add empirical data to the recent discussion about smoothing in functional neuroimaging. Stelzer et al. [31] suggested that smoothing fMRI data should be abandoned altogether, because it (1) causes incorrect estimation of the true spatial extent of brain activation, (2) blurs away signals of limited spatial extent, and (3) frustrates the detection of low-intensity signals in the vicinity of non-active tissue. Our results illustrate quantitatively how large these effects can be, specifically for subcortical nuclei: we show that reported MNI coordinates largely non-overlap with anatomical masks (point 1) and that smoothing can induce substantial mixing with signal from outside the nucleus (point 2 and 3).

The use of smoothing can increase the signal-to-noise ratio in fMRI when the signal is more spatially correlated than the noise on the scale of the smoothing kernel employed. However, in the case of subcortical nuclei, the used smoothing kernels are often too large and mix in signal and noise from neighbouring structures. Yoon et al. [8] provide an empirical example of the influence of kernel size in their supplemental information: the activity in the SN only reached a significance threshold when a smoothing kernel with a very minor FWHM of 2 mm was employed. When a smoothing kernel with an FWHM of 8 mm was used, the effect disappeared. Because the voxel size of this study was rather large, 3.4x4x4 mm, the effect of a 2 mm smoothing kernel was negligible and could have been abandoned altogether. When one applies such a relatively large smoothing kernel to data of such a relatively coarse resolution, the amount of signal in a voxel in the smoothed image originating from outside this voxel is less than 0.2% (See http://nbviewer.ipython.org/gist/Gilles86/0c093962de8cf05f76c8). The results by Yoon et al. thus clearly show that smoothing is not necessary to find significant effects in the substantia nigra region, even with 1.5T [8].

It has been suggested that a lack of spatial resolution and anatomical specificity could be overcome by using unsupervised clustering algorithms such as principal component analysis (PCA; [42]) or independent component analysis (ICA; [42]). These methods might ‘detect’ the nucleus of interest by exploiting the different covariance structures of the BOLD signal in different nuclei. We think, however, that such an approach is not appropriate. First and foremost, it assumes that the task-related BOLD activity in the STN and SN are uncorrelated. This is highly unlikely because both nuclei are part of the same functional networks, e.g., the basal ganglia motor control loops. Secondly, even if the signal could be separated to some extent, there is no objective way of finding out which cluster component belongs to which nucleus and to which extent they account for only one nucleus. Third, independent components might represent non-BOLD signals such as physiological noise. Fourth, the most adequate procedure of defining the actual signal of the nucleus of interest by means of, e.g. a demixing matrix (e.g., [43] or a Gaussian sphere [44]) remains elusive.

Therefore we suggest that during functional imaging of small subcortical nuclei, standard smoothing strategies should be avoided altogether. More complex, adaptive smoothing approaches [45] might be useful, but analysis protocols that do not require smoothing should be preferred. A-priori ROI analyses [46] do not require smoothing, nor do whole-brain univariate analysis approaches that make use of False Discovery Rate (FDR) as multiple comparison correction, as well as multivariate analysis strategies [31,47].

Concretely, we propose an approach that maximizes both anatomical specificity and signal-to-noise. Researchers are advised to use individual anatomical masks based on an appropriate MR contrast (i.e., T2* or quantitative susceptibility mapping (QSM)) that allows for detailed visibility and segmentation of the structures of interest (see, e.g., [27–29,48–50]). When individual segmentation is not feasible, researchers can use probabilistic atlas maps, as provided for the STN and SN in [27–29]. If the research question does not focus on anatomical patterns within the nucleus itself, the mean signal across all voxels in the nucleus can be analysed. This maximizes SNR and removes both the multiple comparisons problem, as well as the need for registration to a standard space. When different activation patterns within the nucleus are expected, a voxel-wise analysis within the anatomical mask can be computed.

Given the variability in reported coordinates and smoothing, one may question the validity of earlier fMRI findings in the STN/SN. It is important to note that studies with Parkinson Disease patients using deep-brain stimulation (DBS) [51] or lesioning of the STN [52] deliver important causal evidence for the functional role of the STN in motor control. fMRI studies that report BOLD activity in motor control paradigms are thus likely to be sensitive to actual task involvement. However, we believe that caution is warranted in interpreting the anatomical specificity of these findings. Especially interpreting findings from studies that 1) use smoothing kernels with a FWHM of more than 4 mm, 2) do not use anatomical masks that are based on individual anatomy, either individually segmented or based on a population probabilistic map like the ATAG dataset [28], and finally, 3) use voxel resolutions that are in the same order of magnitude as the nucleus itself.

In sum, the present study provides evidence for the importance of accounting for individual anatomy when attempting to understand the functional role of small subcortical areas such as the STN and SN. Moreover, the combination of ultra-high resolution fMRI with a very high voxel resolution and zoomed-in acquisition protocols will help to unmix signals arising from small subcortical structures in very close proximity. Finally, the simulation results indicate that spatial smoothing should be avoided when one values anatomical specificity of functional neuroimaging results.
